# Association Between Circulating Cytokines and Endometriosis: A Mendelian Randomization Study

**DOI:** 10.1111/jcmm.70532

**Published:** 2025-04-10

**Authors:** Xiao Xu, Jie Mei, Bin Zhang, Xi‐Ya Jiang, Li Wang, Ai‐Xi Zhang, Jie‐Jie Li, Shun‐Xia Chen, Yu‐Feng He, Ya‐Xing Fang, Lan Zheng, Qin‐Qin Jin, Jing‐Jing Hu, Shu‐Guang Zhou

**Affiliations:** ^1^ Department of Gynecology Maternal and Child Health Center of Anhui Medical University, the Fifth Affiliated Clinical College of Anhui Medical University Hefei Anhui China; ^2^ Department of Gynecology Hefei Maternity and Child Healthcare Hospital, Anhui Women and Children's Medical Center Hefei Anhui China; ^3^ Department of Gynecology Linquan Maternity and Child Healthcare Hospital Fuyang Anhui China; ^4^ Department of Scientific Research Hefei Maternity and Child Healthcare Hospital Hefei Anhui China; ^5^ Department of Clinical Laboratory Linquan Maternity and Child Healthcare Hospital Fuyang Anhui China; ^6^ Department of Public Health Linquan Maternity and Child Healthcare Hospital Fuyang Anhui China; ^7^ Department of Reproduction The First Affiliated Hospital of Anhui Medical University Hefei Anhui China

**Keywords:** circulating cytokines, endometriosis, Mendelian randomization, WGCNA

## Abstract

Existing evidence shows the importance of circulating cytokines in studying female reproductive system dysfunction. Endometriosis (EM) is thought to be associated with multiple immune cytokines, but its causality has not been proven. Utilising Genome‐Wide Association Study (GWAS) data, we performed Mendelian randomisation (MR) to assess causality between 41 cytokines and EM. Positive Single Nucleotide Polymorphisms (SNPs) were annotated via Multi‐marker Analysis of GenoMic Annotation (MAGMA) and intersected with EM‐associated genes from Weighted Gene Co‐expression Network Analysis (WGCNA). Shared genes underwent single‐gene Gene Set Enrichment Analysis (GSEA). The association of shared genes with endometriosis was validated by the quantitative Real‐Time Polymerase Chain Reaction (qRT‐PCR) method. Two‐sample MR identified TNF‐Related Apoptosis‐Inducing Ligand (TRAIL) as causally linked to EM. Inverse variance weighting (IVW) revealed that elevated TRAIL levels reduced EM risk (*β* = −0.061, *p* = 2.267e‐6). WGCNA identified DSG 2 (a TRAIL‐related gene related to EM). Quantitative analysis based on clinical samples confirmed the low expression of DSG 2 in patients with endometriosis. GSEA indicated DSG 2 participation in many signalling pathways. MR analysis revealed that elevated TRAIL levels significantly reduce the risk of EM. MAGMA and WGCNA analyses identified DSG 2 as a key gene associated with TRAIL. Gene expression analysis combined with GSEA suggested that decreased DSG 2 expression may influence the development of EM through various pathways. These results offer new potential diagnostic markers and therapeutic targets for EM.

## 1 Introduction

Endometriosis (EM) is a chronic inflammatory disorder characterised by ectopic endometrial‐like epithelium and/or stroma outside the uterine cavity, typically accompanied by a pelvic inflammatory microenvironment [1]. As a leading cause of gynaecological morbidity, EM manifests clinically as progressive dysmenorrhea (70%–80%), infertility (30%–50%) and multi‐organ pelvic lesions [2, 3]. Notably, 20%–25% of patients are asymptomatic, significantly delaying diagnosis [4]. Globally, EM affects over 170 million reproductive‐aged women, with an estimated population prevalence of 5%–10% [5, 6]. Epidemiological data reveal marked ethnic disparities: Asian women demonstrate the highest risk (6.8%–15.7%), 1.63‐fold and 2.1‐fold higher than African‐American and white women, respectively [1, 6]. These variations may stem from polygenic interactions, differential environmental exposures and systemic healthcare access disparities [6]. Recent European cohorts further report a rising incidence (16.1 per 10 million women, 2014–2017), suggesting escalating disease burden [3, 7]. In this context, in‐depth research into the pathogenesis of EM has become imperative. Multiple hypotheses have been proposed to explain the pathogenesis of EM, yet no single theory fully accounts for all clinical manifestations, pathological features and rare special cases [8, 9, 10]. The retrograde menstruation theory proposed by J.A. Sampson in 1925, which posits that endometrial cells detach from the uterine cavity and implant in the peritoneal cavity, remains the dominant hypothesis [11, 12, 13, 14]. However, this theory struggles to explain why only a small proportion of individuals develop EM, particularly regarding deep/extra‐abdominal lesions [9, 12, 15, 16]. In fact, a large body of current research indicates that the pathogenesis of EM involves multifactorial interactions, including immune dysfunction, genetic predisposition, environmental factors, as well as dietary habits and lifestyle influences [17, 18, 19].

With the deepening of research on EM, it has been found that EM is consistently closely associated with chronic inflammation. A leading theory posits that the body's immune system misidentifies benign ectopic endometrial cells as foreign invaders, triggering chronic inflammation. If the inflammatory clearance of ectopic cells is interrupted during lesion formation, it manifests as subclinical inflammation. However, when anti‐inflammatory mechanisms are insufficient or immune surveillance is impaired, lesions may persist and progress into EM [13, 19]. The causal relationship between local inflammation and the pathogenesis of EM warrants further investigation. Studies have proposed that EM fundamentally manifests as a form of pelvic inflammation analogous to ‘sterile peritonitis’ [20], with abnormal concentrations of inflammatory mediators further supporting inflammation's critical role in disease progression [21]. Peritoneal inflammation coupled with immune dysregulation may jointly contribute to EM development: Impaired immune surveillance permits ectopic endometrial cell survival, but disease establishment ultimately depends on completed adhesion and proliferation processes. This inflammatory–immune microenvironment provides necessary conditions for cellular adhesion, explaining why retrograde menstruation does not invariably lead to EM [22, 23]. At a molecular level, EM lesions exhibit elevated inflammatory mediators (COX‐2, IL‐1β, IL‐8, TNF‐α, PGE2 and E2) compared to normal endometrium [24]. IL‐1β enhances COX‐2 expression, driving PGE2 overproduction, which in turn stimulates E2 synthesis to amplify local inflammation [25]. These interactions establish a positive feedback loop that perpetuates inflammatory responses and immune dysregulation in ectopic tissues [22, 25]. Clinical studies also support these conclusions; studies reveal significantly higher mRNA levels of IL‐6, IL‐8, IL‐1β and TNF‐α in ectopic endometrial lesions compared to normal endometrium, while key Th1‐related cytokines (e.g., IFN‐γ) and cytotoxic activities of NK and T cells are downregulated or dysregulated [26]. This suggests that heightened local inflammation in ectopic lesions reduces cytotoxicity and enhances immunosuppression. Local histiocytes further shape the immune microenvironment by promoting pro‐inflammatory cytokines and suppressing anti‐inflammatory factors [27]. Among them, IL‐6 significantly regulates the survival and proliferation of ectopic endometrial cells [28], and increased cytokines like TNF‐α and interleukins may facilitate peritoneal implantation by disrupting local adhesion mechanisms [29]. Additionally, exosomes released from endometriotic lesions express MICA/B, ULBP1‐3, FasL and TRAIL, which suppress the NKG2D cytotoxic pathway and induce lymphocyte apoptosis to promote immune evasion [30]. Although current evidence underscores EM as a cytokine‐associated chronic inflammatory disease, the specific causal relationship remains unclear. The interaction of chronic inflammation‐driven angiogenesis, hormonal changes and changes in the immune system complicate the comprehensive elucidation of the pathophysiological mechanisms of EM [31]. Moreover, the imbalance between oxidative stress (OS), reactive oxygen species (ROS) and antioxidants induces peritoneal inflammatory responses, which may play a pivotal role in EM pathogenesis [18, 32]. Influenced by inflammatory response and local inflammatory infiltration, EM often presents non‐specific pain symptoms [8]. Given the striking disparities in the clinical manifestations and progression of EM, coupled with the inadequate sensitivity and specificity of imaging examinations, the incidence of delayed diagnosis remains very high [9]. An investigation from Europe, Australia, China and the United States indicated a delay in the diagnosis of EM ranging from 3.5 to 13 years [10, 11, 12, 13, 14]. Delayed diagnosis hinders timely awareness and early treatment of EM. A definitive diagnosis of EM typically requires laparoscopic examination, which is not only invasive but also carries certain risks [15]. Thus, developing a non‐invasive test for EM patients is urgently needed. Unfortunately, among the many EM biomarkers found and proposed in peripheral blood and endometrium to date, none have been clinically validated for their diagnosis [16]. Previous studies have shown that cytokines are involved in pain oogenesis, ectopic implantation and angiogenesis through multiple mechanisms of EM. For example, IL‐8 and IL‐12 affect and regulate the oxidative stress process through participation in the above processes, but the significance, specificity and sensitivity of these cytokines need to be verified [8, 17]. Therefore, we hope to deepen our understanding of EM mechanisms by studying the causal relationship between circulating cytokines and EM, and expect to identify some new biomarkers that may be used for the clinical diagnosis of EM.

Mendelian randomization (MR) effectively addresses confounding factors and reverse causality in traditional observational studies, providing relatively unbiased causal inference [33]. As genetic variants are randomly assigned during fertilisation and unaffected by postnatal environmental factors, MR enables accurate assessment of causal relationships between cytokines and EM [34]. This method allows concurrent evaluation of multiple exposure–outcome interactions, analogous to conducting multiple randomised controlled trials [35]. Previous MR studies have identified 11 inflammatory factors, including monocyte chemotactic protein‐1, macrophage inflammatory protein‐1α and granulocyte colony‐stimulating factor, that show causal associations with EM and may participate in its pathogenesis. Additionally, bioinformatics analyses revealed 568 differentially expressed genes involved in pro‐inflammatory signalling pathways in EM [36, 37].

In this study, we integrated MR with weighted gene co‐expression network analysis (WGCNA) to investigate cytokine‐related genetic mechanisms in EM pathogenesis. Utilising the most comprehensive cytokine dataset from the NHGRI‐EBI GWAS Catalog (41 cytokines) [38], which includes Interleukin‐1, Interleukin‐6, Interleukin‐17 and VEGF (all of which were previously associated with EM [39, 40]), we performed two‐sample MR analysis to establish causality. MAGMA gene set analysis was employed to annotate instrumental variables (IVs) based on SNP physical locations. The WGCNA‐identified EM‐associated genes were intersected with MAGMA‐annotated genes, revealing shared genetic features between cytokines and EM.

## 2 Materials and Methods

### 2.1 Study Design

Using the two‐sample MR analysis, we assessed the causality between 41 cytokines and EM. Three fundamental presumptions must be met by the IV used in the MR analysis of causal inference: (1) relevant assumptions that genetic variation and exposure are directly correlated; (2) independent assumptions that genetic variation and potential confounders between exposure and outcome are related; and (3) exclusive assumptions that the genetic variation will not influence the outcome by any methods other than exposure [18]. GWAS data on positive cytokines were genetically annotated by MAGMA, and the gene signature of EM was identified using WGCNA.

**FIGURE 1:**
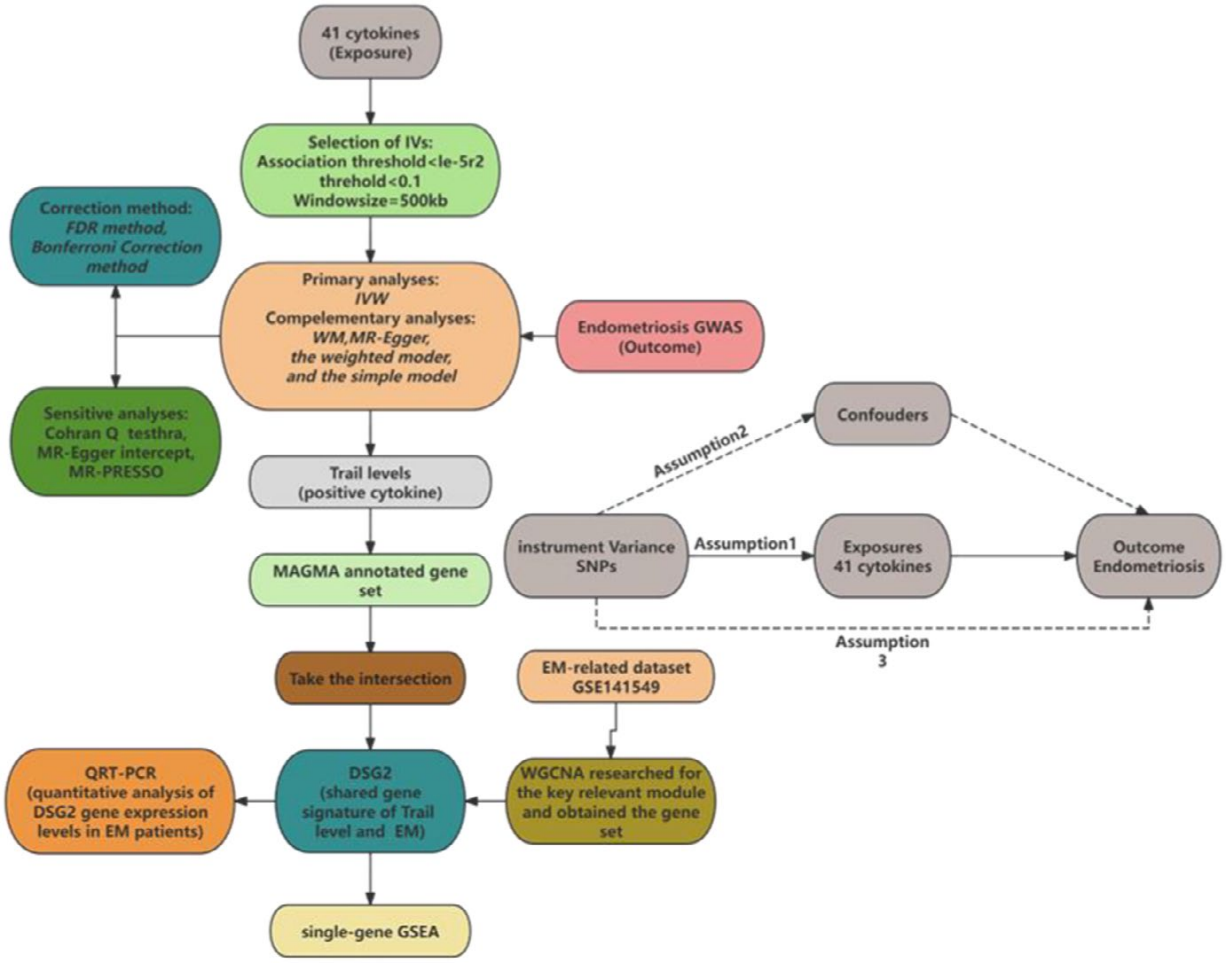
The entire procedure of our study. Assumption 1: The correlation hypothesis: Genetic variation is directly related to exposure; Assumption 2: The independence hypothesis: There is no connection between the genetic variant and the possible confounders between exposure and outcome; Assumption 3: The exclusive hypothesis: The genetic variation will not affect the outcome by means other than exposure. EM, endometriosis; GSEA, Gene Set Enrichment Analysis; GWAS, Genome‐Wide Association Study; IVs, instrumental variables; MR, Mendelian randomisation; SNPs, single nucleotide polymorphisms; WGCNA, weighted gene co‐expression network analysis; WM, the weighted median.

The intersection of the above gene sets was identified as shared gene signatures for cytokines and EM and subjected to gene expression analysis and single‐gene Gene Set Enrichment Analysis (GSEA) [19]. The whole process can be seen in the flow chart in Figure 1.

### 2.2 Data Source

In this study, we utilised data from a large‐scale, published genome‐wide association study (GWAS) meta‐analysis encompassing 41 cytokines, representing the most comprehensive dataset available at the time (PMID: 27989323). Some cytokines in this dataset, like IL‐1, IL‐6, IL‐17 and vascular endothelial growth factor (VEGF), were associated with EM pathogenesis (PMID: 39110084, PMID: 38466035), justifying the selection of these 41 cytokines as a representative panel.

GWAS summary statistics for circulating cytokines were derived from prior research [20], including the Cardiovascular Risk in Young Finns Study (YFS) and FINRISK cohorts. Individual cytokine GWAS data are accessible via the GWAS Catalog (accession numbers GCST004420‐GCST004460; Appendix S1 and S2). EM‐related GWAS summary statistics were obtained from the FinnGen Consortium Release 9 (R9), comprising 15,088 cases and 107,564 controls (all Finnish females) [21]. Detailed exposure and outcome data are provided in Table 1.

**TABLE 1:** Details of GWAS summary data.

GWAS data	Population	Source	Ncase	Ncontrol
41 cytokines	European	Ahola‐Olli et al. (https://www.ebi.ac.uk/gwas/publications/32929287)	8293	—
finngen_R9_N14_ENDOMETRIOSIS	European	FinngenR9 (https://storage.googleapis.com/finngen‐public‐data‐r9/summary_stats/finngen_R9_N14_ENDOMETRIOSIS.gz)	15,088	107,564

We performed WGCNA for EM using the GSE141549 dataset from the Gene Expression Omnibus (GEO; http://www.ncbi.nlm.nih.gov/geo). This human‐derived dataset (platform GPL13376) initially included 338 endometrial biopsies (43 controls, 295 EM patients). After quality control, 133 ectopic endometrial samples were retained for analysis [22].

### 2.3 Selection of Instrumental Variables (IVs)

To satisfy the correlation hypothesis of the Mendelian assumption, we set the significance level for cytokines to 1e‐5 and used PLINK (version 1.90) to exclude SNPs within a 500 kb range with an associated coefficient *r* ^2^ threshold of 0.1 [23]. Subsequently, we utilised the MR‐Egger intercept and MR‐PRESSO methods to identify and rule out multi‐effectiveness abnormalities [24].

### 2.4 Mendelian Randomisation

The R package ‘TwoSampleMR’ (version 0.1.5.6) in the R 4.2 program was applied to carry out our MR analysis. Inverse variance weighting (IVW), the weighted median (WM), the simple model, MR‐Egger and the weighted model included in the MR analysis were used to investigate the causality between exposure and outcome. The crucial method for identifying causal effects is IVW, which offers the highest precision and unbiased causal estimates [25]. The remaining four analytical methods act as auxiliary methods to assure the robustness of the results [26]. After that, we performed a sensitivity analysis using Cochran's *Q* based on IVW to assess heterogeneity (> 0.05 was deemed nonsignificant), and we evaluated the dependability of the heterogeneity results using funnel plots [27]. We tested for horizontal pleiotropy using the MR‐PRESSO and MR‐Egger intercept approaches in order to satisfy the MR exclusivity hypothesis [24]. Taking into account the existence of false positives, we adjusted the *p*‐values for the positives. Using the Bonferroni correction approach, we determined that circulating cytokines had a significant causal connection according to *p* < 0.0012 [28].

### 2.5 Gene Annotation Analysis Based on Chromosomal Physical Positions

The chromosome (Chr) and position (Pos) were collated in the GWAS data of positive results based on the Reference SNP ID (rsID) of SNPs. Using MAGMA, the SNPs correspond to the corresponding gene according to the physical position on the chromosome; 36 genes were identified in total. The MAGMA and gene annotation files were downloaded at https://cncr.nl/research/magma.

### 2.6 Gene Predictive Analysis

MAGMA identified a gene set related to cytokines by annotating their IVs. The WGCNA R program was utilised for analysing the expression data from the GSE141549 dataset. Co‐expression networks were created by combining the clinical features of ectopic lesion samples from EM patients and endometrial tissues from healthy individuals.

By utilising the ‘pickSoftThreshold’ function of the WGCNA package, the ideal filtering power value for the soft threshold was calculated. The power value brings the scale‐free network structure *R* ^2^ to 0.8 for the first time, and then the one‐step co‐expression matrix was generated utilising the ‘blockwisemomodules’ function. The adjacency of expression levels across all genes was computed to create the adjacency matrix [29]. The adjacency matrix was then transformed into a topological overlap measure (TOM) matrix so that its connectivity property inside the network could be assessed. In order to obtain appropriate modules, the minimum gene module size was set to 30 when utilising the corresponding difference degree (1‐TOM) as the measurement.

In addition, the threshold for merging similar modules was set to 0.25. The module with the strongest connection to the EM (|correlation coefficient| > 0.5, *p* < 0.05) was isolated [30]. Screening out key module genes for intersection with the MAGMA gene set yielded EM‐related genes in inflammatory factor‐related genes, which were subsequently subjected to single‐gene GSEA. The threshold for the significance of the enrichment was the NOM *p* < 0.05.

### 2.7 Collection of Human Tissues

From September to October 2024, we recruited a total of 10 patients at the Department of Gynaecology, Hefei Maternal and Child Health Care Hospital Linquan Branch. The experimental group comprised five specimens of endometriotic tissues obtained from ovarian chocolate cysts, while the control group included five endometrial tissues collected during myomectomy procedures for uterine fibroids.

### 2.8 qRT‐PCR for Shared Genes Validation

Quantitative reverse transcription PCR (RT‐qPCR) was performed to compare DSG 2 expression levels between ectopic lesions from five endometriosis patients (study group) and eutopic endometrial tissues from five uterine fibroid patients (control group).

Following their freezing in liquid nitrogen, total RNA was extracted from five endometriosis and five normal specimens applying the TRIzol reagent (15,596,018, America). Reverse transcription was carried out at 42°C for 15 min, followed by 3 min at 95°C. The Talent qPCR PreMix was then employed in a 10 μL SYBR reaction mixture. The protocol included 40 cycles at 95°C for 5 s and at 60°C for 15 s, coupled with an initial cycle at 95°C lasting 3 min. Then, the desired sequences of mRNA with the best melting curves and sizes were found. Table 2 shows primer sequences that were employed in the PCR process.

**TABLE 2:** PCR primer information.

Primer name	Primer sequence(5′ → 3′)	Amplification product (bp)
H‐DSG‐2‐F	TCTTCTAGGCAGGCGCAAAA	112
H‐DSG‐2‐R	TGGTTGGTGGCATAGTGGAC	
h‐FOS‐F	GGGGCAAGGTGGAACAGTTAT	126
h‐FOS‐R	CCGCTTGGAGTGTATCAGTCA	
H‐THBS1‐F	TGCTATCACAACGGAGTTCAGT	108
H‐THBS1‐R	GCAGGACACCTTTTTGCAGATG	
H‐PLA2G2A‐F	GAAAGGAAGCCGCACTCAGTT	122
H‐PLA2G2A‐R	CAGACGTTTGTAGCAACAGTCA	
H‐CEACAM1‐F	AACCAAAGCGACCCCATCAT	195
H‐CEACAM1‐R	GTGCTCTGTGAGATCACGCT	
H‐GAPDH‐F	GGAGCGAGATCCCTCCAAAAT	197
H‐GAPDH‐R	GGCTGTTGTCATACTTCTCATGG	

## 3 Results

### 3.1 Exploration of the Causality Between Cytokines and EM

**FIGURE 2:**
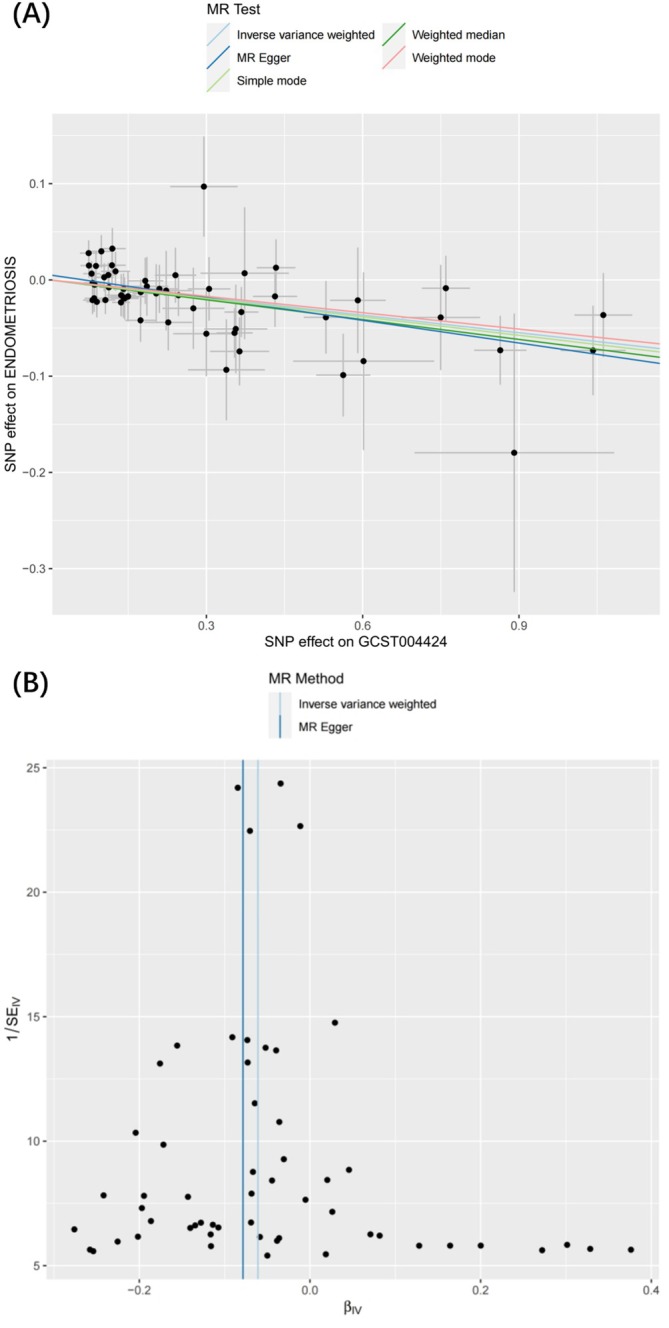
Scatter plots and funnel plots illustrating the causal associations between cytokines and endometriosis. (A) Scatter plots of TRAIL on ENDOMETRIOSIS; (B) Funnel plots of TRAIL on ENDOMETRIOSIS.

We performed a two‐sample MR analysis using 41 cytokines as exposure factors. Subsequently, the data were corrected using the Bonferroni method to exclude false outcomes (adjusted significance threshold: *p* < 0.0012, calculated as 0.05/41). Using the IVW analysis method, we observed that a lower risk of EM can result from higher *TRAIL* levels (*β* = −0.061, 95% CI = 0.92–0.96, *p* = 2.267e‐6) and similar results of the other two analyses, validating the reliability of the results: Weighted median (*β* = 0.069, 95% CI = 0.90–0.97, *p* = 4.426e‐4); MR‐Egger (*β* = −0.079, 95% CI = 0.89–0.99, *p* = 1.412e‐4) (Appendix S3). Scatter and funnel plots supported the reliability of these findings (Figure 2), though funnel plot asymmetry should be interpreted cautiously. The results showed no significant heterogeneity or horizontal pleiotropy (Table 3), further validating robustness.

**TABLE 3:** MR analysis results of causal relationships among cytokines and endometriosis.

Exposure	SNPs	Method	OR (95% CI)	*p*	Heterogeneity			Horizontal pleiotropy Intercept P	MR‐PRESSO
					*Q*	*Q*_df	*p*		*p*
Trail levels	56	IVW WM ME	0.94 (0.92–0.96) 0.93 (0.90–0.97) 0.92 (0.89–0.96)	2.267E‐6 0.0004 0.0001	51.88	55	0.5944	0.2216	0.6260

### 3.2 Predictive for Shared Gene Features of TRAIL and EM

**FIGURE 3:**
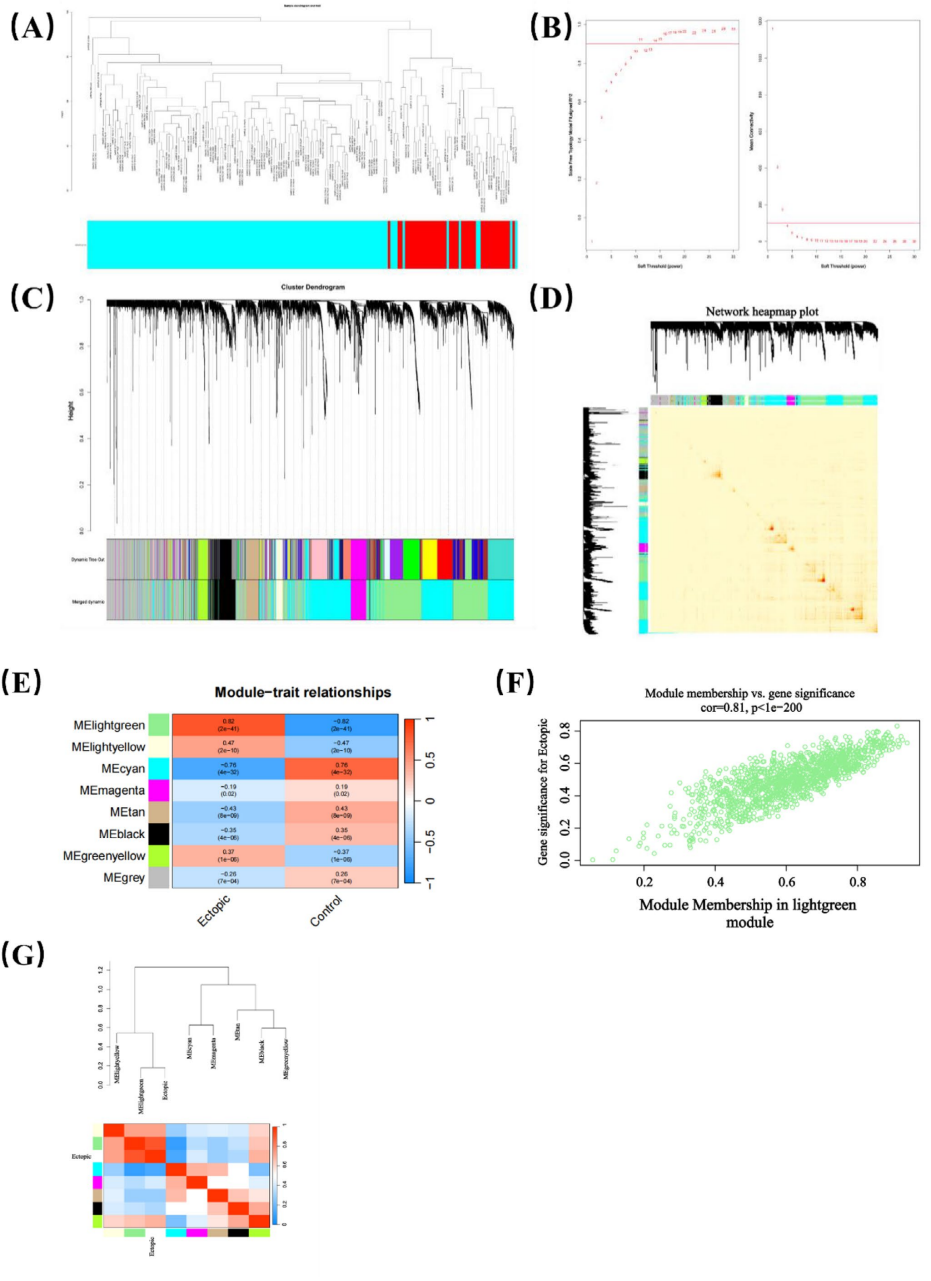
WGCNA screened out the modules most relevant to endometriosis. (A) Preliminary cluster analysis; (B) Analysis of network topologies for various soft‐threshold powers; (C) Dendrograms of genes with different similarity based on module colours of topological overlap and assignments; (D) Heatmap view of co‐expressed genes in different modules. (E) Correlation between eight key modules and clinical traits; (F) GS versus MM in the lightgreen module. (G) Correlation of modules and ectopic.

**FIGURE 4:**
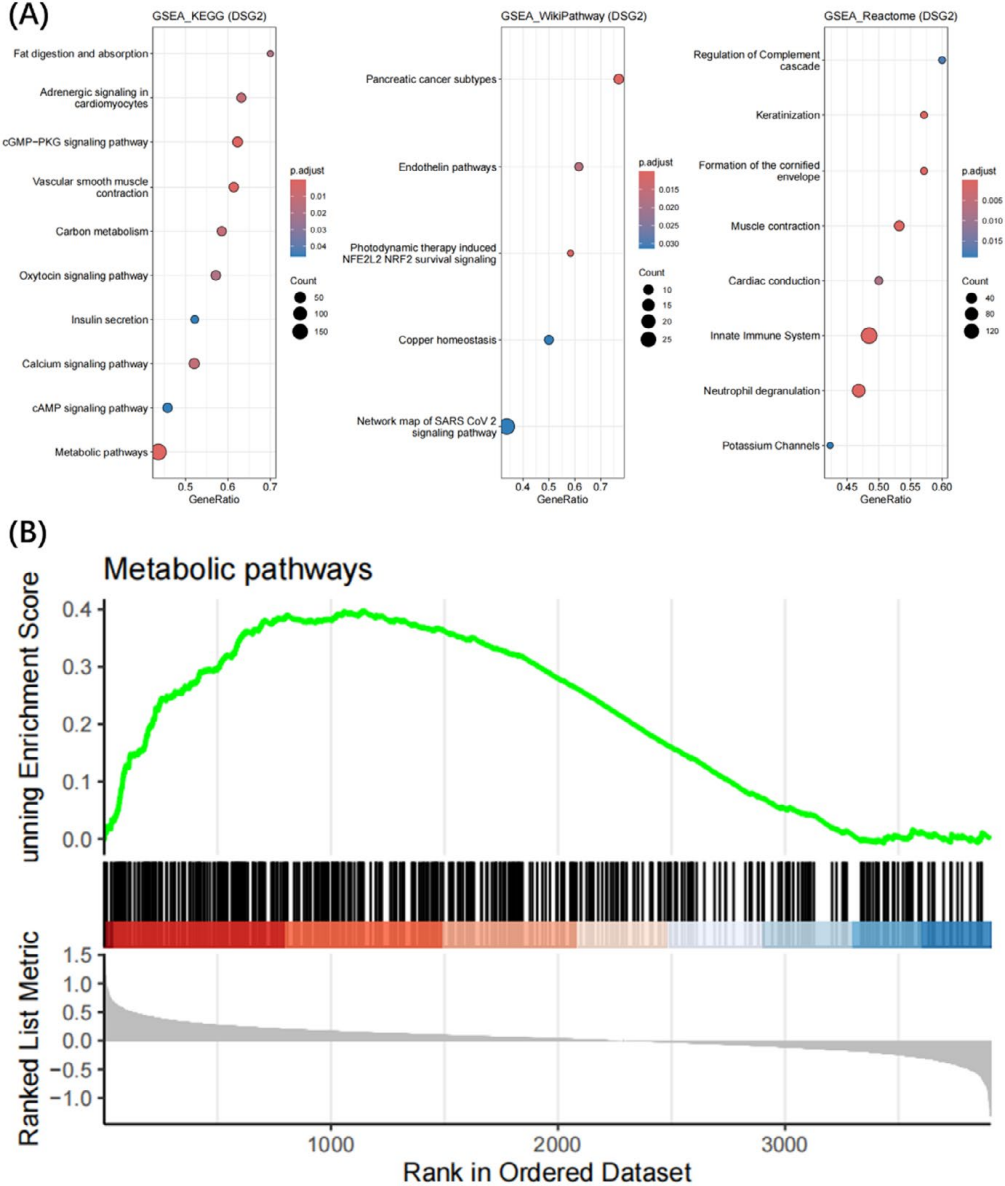
The single‐gene GSEA of DSG2. (A) GSEA pathway analysis based on the three databases. (B) Metabolic pathway enrichment analysis.

Based on MAGMA, 21 genes were identified by gene set analysis of GWAS genotype data, which can be found in Appendix S4. Using WGCNA, we explored the genes related to EM in the GSE141549 dataset. The WGCNA networks were constructed using the 5000 genes that had the highest median absolute values. Cluster analysis removed samples that had significant outliers and false clusters (Figure 3A). After selecting the optimal soft threshold of 11 (Figure 3B), the genes were divided into multiple modules according to the dynamic tree species cutting method (Figure 3C). Then, 0.25 was selected as the threshold for merging the relevant modules into eight modules, including the black module (341 genes), the cyan module (1563 genes), the green‐yellow module (173 genes), the grey module (1105 genes), the light‐green module (1335 genes), the light‐yellow module (92 genes), the magenta module (223 genes) and the tan module (168 genes). We then calculated the association of the modules with the clinical features. As shown, the light green module has the largest positive association with the EM (cor = 0.82, *p* = 2 e‐41) (Figure 3D,E). In order to verify the correlation between light green modules and gene significance, we performed a comprehensive calculation. The results show that the light green module is closely related to the EM (cor = 0.81, *p* < 1e‐200) (Figure 3F,G). Next, after intersecting the gene sets of the light green module and MAGMA annotated gene sets, we identified DSG2 as a shared gene signature of cytokines and EM. Single‐gene GSEA of DSG2 showed that DSG2 participates in various pathways including metabolic pathways and cGMP PKG signalling pathway (Figure 4).

### 3.3 Expression Profiling of DSG 2 in Endometriotic Lesions

**FIGURE 5:**
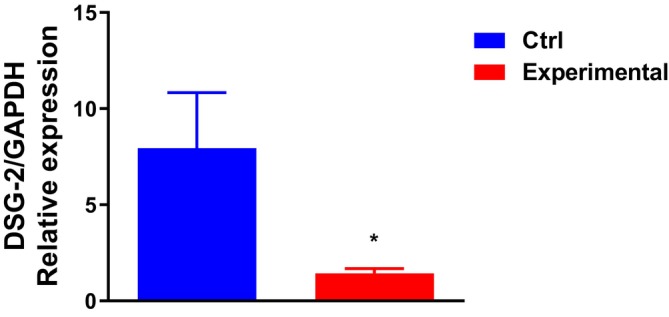
The single‐gene quantitative expression analysis of DSG2. The relative expression level of DSG2 in the Ctrl group and the Experimental group.

The quantitative expression analysis of DSG2 gene in ectopic endometrial tissue (Experimental group) and normal endometrial tissue (Ctrl group) using RT‐PCR revealed significant differences between the two groups. After normalisation with the reference gene GAPDH, the relative expression level of DSG2 in the Ctrl group was approximately 8 (mean value), while it was markedly reduced to nearly 1 in the Experimental group (*p* < 0.05) (Figure 5). This indicates that the transcriptional level of DSG2 is significantly suppressed in ectopic endometrium, with statistically meaningful differences.

## 4 Discussion

Mendelian randomisation is a statistical analytic method to infer causality based on genetic characteristics and has recently been extensively applied in etiological studies [31]. This study provides statistical evidence to explore the causal relationship between 41 circulating cytokines and EM. By using large MR analysis based on the public GWAS database, we revealed the potential correlation between TRAIL (as a cytokine) and EM pathogenesis by constructing a WGCNA and identifying shared gene characteristics between TRAIL and EM. This approach may provide new insights into EM aetiology. Furthermore, partial findings are supported by clinical evidence. A prospective cohort study involving 81 patients (18–50 years old), including 51 EM patients (18 with deep‐infiltrating endometriosis, DIE) and 30 controls, analysed protein levels in plasma and peritoneal fluid. TRAIL levels were significantly lower in EM patients compared to controls in both plasma and peritoneal fluid (*p* < 0.05) [32]. These results support the hypothesis that reduced peripheral blood TRAIL levels correlate with increased EM risk [41]. Tumour necrosis factor‐related apoptosis‐inducing ligand (TRAIL), a member of the TNF superfamily, selectively induces apoptosis in transformed or malignant cells by binding to its cognate death receptors DR4 (TRAIL‐R1) and DR5 (TRAIL‐R2). This interaction activates the extrinsic apoptotic pathway via caspase‐8/10‐mediated cleavage of downstream effectors (e.g., caspase‐3), culminating in programmed cell death. TRAIL signalling is tightly regulated by decoy receptors (DcR1, DcR2), which competitively inhibit apoptosis by sequestering TRAIL, and by transcriptional modulators such as NF‐κB, STAT3 and interferon‐responsive elements. Notably, interferons (IFN‐γ, IFN‐α/β) upregulate TRAIL expression, while inflammatory cytokines (e.g., IL‐6) suppress it through STAT3/PI3K‐dependent pathways [41, 42]. Based on the known biological role of TRAIL factors, we propose that dysregulation of TRAIL signalling may contribute to endometriosis (EM) pathogenesis. A hallmark of EM is the resistance of ectopic lesions to apoptosis, partly attributed to an imbalance in TRAIL receptor expression. Specifically, EM lesions exhibit reduced expression of pro‐apoptotic death receptors DR4 and DR5 alongside elevated levels of the anti‐apoptotic decoy receptor DcR1. This imbalance disrupts TRAIL‐mediated apoptosis, enabling ectopic cell survival. In murine EM models, TRAIL mRNA levels are significantly reduced in ectopic tissues, correlating with increased DcR1 expression and neutrophil infiltration [32, 42]. Clinical studies further corroborate these findings: Neutrophils from EM patients show reduced TRAIL protein levels alongside overexpression of MMP9 (Matrix Metalloproteinase‐9). This dual effect is likely driven by pro‐inflammatory cytokines such as G‐CSF (Granulocyte Colony‐Stimulating Factor) and IL‐6 (Interleukin‐6), which suppress TRAIL transcription via the STAT3/PI3K (Signal Transducer and Activator of Transcription 3/Phosphatidylinositol 3‐Kinase) pathway while upregulating MMP9 [32, 41]. Genetic variations in TRAIL receptors may also contribute to apoptotic resistance. For instance, the DR4 rs20576 polymorphism has been associated with altered apoptosis sensitivity in EM stromal cells. Additionally, EM‐related microRNAs, including miR‐21 and miR‐155, directly target components of the TRAIL pathway, enhancing cellular tolerance to apoptosis [43, 44].

According to our gene prediction analysis, DSG2 is a gene characteristic shared by cytokines and EM [45]. The DSG2 gene, located at chromosome 18q12, encodes the calcium‐dependent adhesion molecule Desmoglein‐2 (Dsg2), a core component of desmosomes that regulates mechanical coupling and signalling in cardiac, epithelial and embryonic tissues [38, 46, 47]. Dsg2 is a calcium‐dependent transmembrane glycoprotein belonging to the large cadherin family. It mainly functions as a component of the intercellular desmosome, maintaining the cell–cell adhesion function. However, recent studies have shown that the role of DSG2 in multiple diseases extends far beyond this [45]. In cancer, DSG2 promotes the proliferation, migration and invasion of tumour cells by activating various signalling pathways, such as EGFR, c‐Src, Stat 3, etc. [33]. In cancer correlation studies, the expression level of DSG2 is closely related to the development of tumours. In cervical cancer, DSG2 affects the proliferation, migration and invasion ability of cancer cells by mediating the MAPK signalling pathway, suggesting that it may become a novel target for the treatment of cervical cancer [34]. Notably, DSG2 has an important role not only in cancer but also significant effects in other diseases. For example, in arrhythmogenic cardiomyopathy (ACM), a mutation in the DSG2 gene is associated with mechanical stress and inflammatory responses in the heart, suggesting a potential association of DSG2 in cardiac disease [35]. Moreover, DSG2 is also involved in regulating apoptosis, and in intestinal epithelial cells, DSG2 is cleaved by caspases, thereby affecting the cellular apoptotic process [36, 37]. DSG2 is essential for the proliferation of the inner cell mass and embryonic stem cells during embryonic development, indicating its importance in non‐desmosome‐related functions [38]. Not only that, DSG2 is also involved in the infection process of this virus, such as a human adenovirus, which achieves cell infection by binding to DSG2 [39].

There is currently no direct evidence for a direct association of Dsg 2 with EM pathogenesis, but given the role of Dsg2 in other cancers, especially in regulating cell adhesion and migration, we speculate that Dsg2 may affect EM progression by affecting the adhesion and migration ability of endometrial cells. Desmosomes, a highly controlled protein complex that mediates intercellular adhesion in a calcium‐dependent manner, are encoded by the DSG2 gene [40]. It has been found that alterations in preimplantation desmosomes and cadherin distribution imply less lateral adhesion between epithelial cells, thereby permitting blastocyst implantation [48]. In other words, DSG2 influences blastocyst implantation and has a function in controlling endometrial cell adhesion. We inferred that the process of implanting ectopic endometrial cells into the abdominal cavity might also be regulated by the above mechanisms. Desmosomal cadherins have been demonstrated to play a function in regulating the homeostatic signals from epithelial cells, as well as controlling intercellular adhesion [49]. For example, Dsg2 may maintain endometrial epithelial integrity by forming heterophilic complexes with Desmocollin‐2 (Dsc2), thereby preventing ectopic cell invasion [50]. Additionally, DSG2 might interact with the NF‐κB and STAT3 signalling pathways to suppress the production of pro‐inflammatory cytokines that are overactive in EM, thereby reducing lesion establishment [51]. In cancer, DSG2 promotes vasculogenic mimicry (VM) via upregulation of CEBPD, while its downregulation in EM could inhibit abnormal vascularisation in ectopic tissues [52]. Furthermore, uterine DSG2 expression is oestrogen‐sensitive, suggesting that DSG2 dysregulation may alter oestrogen receptor signalling and enhance endometrial cell proliferation [53]. While these mechanisms are plausible based on DSG2's known roles, direct experimental validation in EM is lacking.

Emerging evidence suggests that TRAIL may interact with DSG2 to regulate endometrial cell behaviour, potentially contributing to its protective role in endometriosis. TRAIL activates the death receptor DR4/DR5, leading to caspase‐8 activation and downstream apoptotic signalling [54, 55]. Notably, it was shown that TRAIL induces lysis of DSG2 into intracellular (ICF) and extracellular (ECF) fragments, similar to TNF‐α and IFN‐γ, where DSG2 ICF production preceded maximum PARP cleavage and mitochondrial cytochrome c release. This fragment sensitises epithelial cells to apoptosis by downregulating the anti‐apoptotic proteins Bcl‐XL and Mcl‐1 (with 50% reduction) and promoting mitochondrial outer membrane permeability [49]. In endometriosis, ectopic endometrial cells evade apoptosis, and TRAIL‐mediated DSG2 proteolysis can enhance the apoptotic clearance of ectopic endometrial cells, thereby limiting the establishment of lesions. In addition, activation by TRAIL or other stimuli (e.g., adenovirus Ad 3/Ad 14 through ADAM17) may disrupt intercellular adhesion and reduce endometrial cell detachment and invasion, a critical step in the pathogenesis of endometriosis [39]. Moreover, TRAIL has anti‐inflammatory effects by inhibiting the NF‐κB or STAT3 pathways and may synergise with the role of DSG2 in maintaining epithelial integrity to mitigate inflammation‐driven lesion growth [49].

Despite the lack of direct evidence for TRAIL's involvement in EM pathogenesis, the overlap between TRAIL‐mediated apoptotic mechanisms and the structural/signalling functions of DSG2 provides a plausible mechanistic framework. Currently, the role of TRAIL in EM pathogenesis and its feasibility as a diagnostic biomarker require further validation. Additionally, the involvement of DSG2 in EM pathogenesis warrants deeper investigation. Future studies should prioritise evaluating TRAIL‐DSG2 axis activity in endometriotic lesions, particularly the correlation between DSG2 cleavage fragments, apoptosis markers and clinical severity.

## 5 Conclusion

In conclusion, our study suggests that both TRAIL and DSG2 most likely have a close causal relationship with EM and may both be involved in EM development through multiple mechanisms. The feasibility of TRAIL as a biomarker for the diagnosis of EM remains to be further investigated and required for clinical validation. The role of DSG2 in the pathogenesis of EM also deserves further study and exploration through a larger sample size and functional experiments.

### 5.1 Strength and Limitation

Our results are robust because no pleiotropy or heterogeneity was established. However, there are still some limitations. The GWAS data samples for the exposure factors in this study were not stratified by sex, while the resulting GWAS samples included only women. This lack of gender stratification may lead to a bias in the results, despite the fact that many identical articles have been published [56]. We did not extract individual SNPs from the positive results using a Pheno Scanner to eliminate any biased SNPs [57]. In addition, the sample we obtained was from a European population. Therefore, additional verification is required to ascertain whether this conclusion holds true for the Asian community [58]. By integrating MR with WGCNA, this study identified a cytokine significantly associated with endometriosis and further explored their shared genetic characteristics. This discovery enhances our understanding of the pathogenesis of endometriosis and provides novel directions for developing clinical diagnostic biomarkers and therapeutic targets. It also offers new insights into investigating the mechanisms underlying complex diseases by revealing immune‐related molecular pathways and gene expression patterns linked to disease progression.

## Author Contributions

**Xiao Xu:** writing – original draft (equal). **Shu‐Guang Zhou:** writing – review and editing (equal). **Jie Mei:** writing – review and editing (equal). **Bin Zhang:** writing – review and editing (equal). **Xi‐Ya Jiang:** writing – review and editing (equal). **Li Wang:** writing – review and editing (equal). **Ai‐Xi Zhang:** writing – review and editing (equal). **Jie‐Jie Li:** writing – review and editing (equal). **Shun‐Xia Chen:** writing – review and editing (equal). **Yu‐Feng He:** writing – review and editing (equal). **Ya‐Xing Fang**: writing – review and editing (equal). **Lan Zheng**: writing – review and editing (equal) **Qin‐Qin Jin**: writing – review and editing (equal). **Jing‐Jing Hu**: writing – review and editing (equal).

## Ethics Statement

Anhui Women and Children's Medical Center Ethics Committee approved the study protocol (Ethical number: YYLL20231021‐YNXM‐LL‐03‐1.0). The experiment has passed the audit of the Chinese Clinical Trial Registry (Registration number: ChiCTR2400088899). All participants were informed of the study procedures and objectives, and this study was conducted after participants signed an informed consent form. This study complied with the Declaration of Helsinki, adhered to ethical guidelines and ensured that participants' rights and confidentiality were protected.

## Conflicts of Interest

The authors declare no conflicts of interest.

## Supporting information


Appendix S1.

Appendix S2.

Appendix S3.

Appendix S4.

## Data Availability

The datasets studied in our research are accessible in the GWAS catalog, GEO database and FinngenR9 version, with download addresses provided in Table 1 and Appendix S1. The data in this study can be made available for use and/or analysis by contacting the corresponding author upon reasonable request.
