# Supplementary figures and images for: Association Between Circulating Cytokines and Endometriosis: A Mendelian Randomization Study
Source: J Cell Mol Med. 2025 Apr 10;29(7):e70532. doi: 10.1111/jcmm.70532 (PMC11984317; doi:10.1111/jcmm.70532)

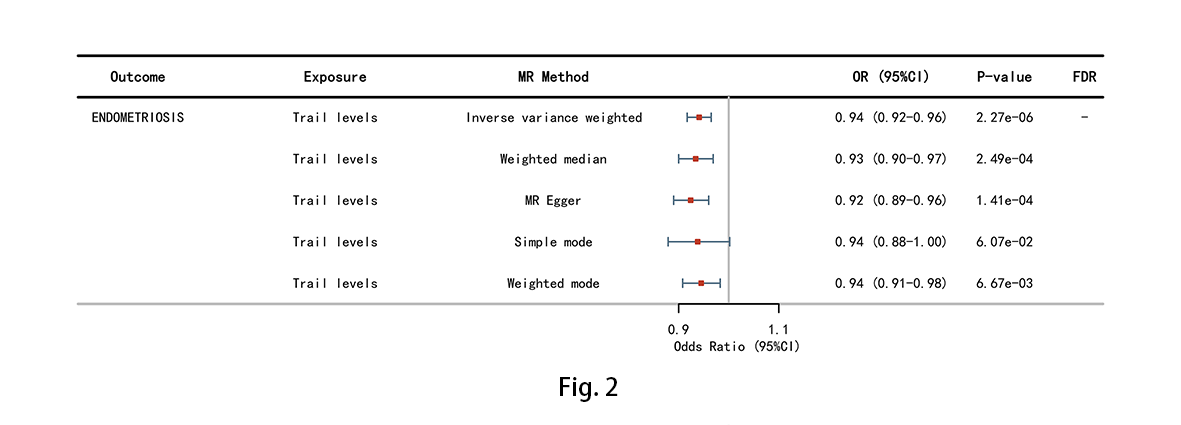

Supplement: Supplementary file 3 — Appendix S3. [file JCMM-29-e70532-s002.tif]
